# Complement C3 deficiency enhances renal leptospiral load and inflammation while impairing T cell differentiation during chronic *Leptospira interrogans* infection

**DOI:** 10.1128/iai.00398-25

**Published:** 2025-11-18

**Authors:** Leonardo Moura Midon, Amaro Nunes Duarte Neto, Ana Maria Gonçalves da Silva, Marcos Bryan Heinemann, Suman Kundu, Maria Gomes-Solecki, Lourdes Isaac

**Affiliations:** 1 Department of Immunology, Institute of Biomedical Sciences, University of São Paulo 28133, São Paulo, Brazil; 2 Department of Pathology, Faculty of Medicine, University of São Paulo 28133, São Paulo, Brazil; 3 Tropical Medicine Institute, Faculty of Medicine, University of São Paulo 28133, São Paulo, Brazil; 4 Bacterial Zoonosis Laboratory, Medicine Veterinary School, University of São Paulo 28133, São Paulo, Brazil; 5 Department of Microbiology, Immunology and Biochemistry, University of Tennessee Health Science Center 12326, Memphis, Tennessee, USA; Rutgers New Jersey Medical School, Newark, New Jersey, USA

**Keywords:** Complement System, leptospirosis, C3, chronic kidney fibrosis, pathology, T cell maturation

## Abstract

Leptospirosis is a neglected disease caused by pathogenic *Leptospira* spp., affecting an estimated 1 million people annually and resulting in approximately 60,000 deaths. The disease can lead to hepatic, renal, and pulmonary dysfunctions and may contribute to the development of chronic kidney disease. The Complement System plays an important role in eliminating bacteria by lysis, generating opsonins and anaphylatoxins, which degranulate mastocytes and basophils, and attracting immune cells to the site of infection, among other important functions. We aimed to investigate the role of C3 during chronic infection by *L. interrogans* strain FIOCRUZ L1-130 (LIC) in C57BL/6 wild-type (WT) and C3 knockout (C3KO) mice, monitored for 15, 30, 60, 90, or 180 days post-infection (d.p.i.). LIC-infected C3KO mice exhibited significantly higher leptospiral loads in the kidneys compared to WT counterparts. While both groups showed local inflammation at 15 and 30 d.p.i., LIC-infected C3KO showed a higher number of *Leptospira* DNA copies at 30 d.p.i. At this same time point, C3KO LIC-infected mice developed a larger fibrotic area than WT mice. Additionally, levels of specific IgG2b and IgG3 antibodies were significantly higher in LIC-infected C3KO mice compared to WT mice. The number of naïve T lymphocytes (both CD4^+^ and CD8^+^) was also increased in LIC-infected C3KO mice. This study demonstrates that during LIC infection, the absence of C3 does not impact mouse survival but results in increased renal leptospiral load and fibrosis. It also highlights the role of C3 in promoting the maturation and differentiation of T lymphocytes into pre-effector cells.

## INTRODUCTION

Leptospirosis is a neglected disease and one of the most important zoonoses worldwide, affecting an estimated 1 million people and causing 60,000 deaths annually (1). It is caused by the pathogenic *Leptospira* spp. (2, 3), a Gram-negative spirochete occurring mainly in developing countries with tropical and mild climates and poor sanitary conditions. In urban centers, infected rodents, such as *Rattus norvegicus* (brown rat), serve as primary reservoirs of *Leptospira*, shedding bacteria through their urine (4, 5).

Humans are accidental hosts who become infected upon contact with contaminated water or soil. *Leptospira* can penetrate the host through the mucosa or damaged skin. While most cases are asymptomatic or present with mild symptoms such as fever, headache, muscle pain, nausea, and vomiting during the blood dissemination phase (the acute phase), these symptoms may be similar to other acute infections, leading to underreporting of leptospirosis (3, 4).

Leptospirosis can lead to multiple organ dysfunction, particularly affecting the liver, kidneys, and lungs (6). In severe cases, such as Weil’s syndrome, complications may include jaundice, kidney and liver failure, internal hemorrhage, and pulmonary distress, which can be fatal (7). Pulmonary hemorrhage associated with leptospirosis is another possible severe manifestation (8), with a high mortality rate (30%–60%) and the potential to cause death in a short period (9, 10). Leptospirosis may also be linked to chronic kidney disease of unknown etiology in endemic areas of Southeast Asia and Latin America (11).

C3 is the most abundant Complement System protein (12) in the serum and plays a key role in activating the Alternative, Classical, and Lectin Pathways, which converge at the Terminal Pathway to generate the membrane attack complex on pathogen surfaces, potentially causing osmotic imbalance and destruction. C3 mediates various immune functions, including (i) attracting immune cells to the site of inflammation via its C3a fragment; (ii) opsonizing pathogens through C3b and iC3b fragments, facilitating phagocytosis via Complement Receptors (CR); (iii) activating mast cells and basophils, leading to release of inflammatory mediators; and (iv) supporting B-lymphocyte activation and antibody production via the C3d fragment (reviewed in references 13, 14). C3 deficiency is associated with susceptibility to severe and recurrent bacterial infections (14, 15), mainly by *Neisseria meningitidis*, Haemophilus influenza, or *Streptococcus pneumoniae* (14, 15), as well as chronic renal diseases, such as membranoproliferative glomerulonephritis (16). Dysregulated activation of C3 can also lead to tissue damage, particularly in the kidney (17–19).

While non-pathogenic *Leptospira* are *in vitro* rapidly killed after the Alternative Pathway is activated in the serum (20–22), pathogenic ones have evolved immune evasion mechanisms to circumvent the Complement System (23) and disseminate in the host by (i) binding to host Complement regulatory proteins (24); (ii) acquiring host proteases capable of cleaving C3 and C5 (25); and (iii) secreting metalloproteases such as thermolysin (26, 27) and leptolysin (28) that cleave Complement proteins and macrophage surface molecules necessary to phagocytosis (29).

In a previous study conducted by our group (22), we investigated the role of C3 during the acute phase of infection with *L. interrogans* serovar Kennewicki strain Pomona Fromm in C57BL/6 wild-type (WT) and C3-deficient (C3KO) mice. The absence of C3 was associated with significantly higher leptospiral loads in the kidneys, liver, spleen, and urine compared to WT mice at 3 and 6 days post-infection (d.p.i.). In addition, C3KO mice exhibited reduced numbers of CD4^+^ and CD8^+^ T lymphocytes in the spleen compared to infected WT mice. Interestingly, interstitial nephritis was observed in C3KO mice at 15 d.p.i., raising new questions about the role of C3 beyond the acute phase of leptospirosis, particularly after kidney colonization has been established.

In the present study, we investigated the role of C3 in a chronic model of leptospirosis using *L. interrogans* serovar L1-130 FIOCRUZ (LIC). We evaluated multiple time points to assess the contribution of C3 to kidney fibrosis development and the immune response against this pathogen.

## MATERIALS AND METHODS

### Animals

Male, 10-week-old C57BL/6J wild-type (WT) (RRID:IMSR_JAX:000664) and B6.129S4-C3^tm1Crr^/J (RRID:IMSR_JAX:029661) congenic homozygous C3-deficient (C3KO) mice were purchased from The Jackson Laboratory (Bar Harbor, ME, USA). Animals were maintained and used in a specific pathogen-free environment at the Animal Care Facilities at the Institute of Biomedical Sciences of the University of São Paulo (ICB-USP) (CEUA number: 9917191218) or at the University of Tennessee Health Science Center (UTHSC) (committee protocol number: 22-0362), as indicated in each figure legend.

### *Leptospira* infection

Pathogenic *L. interrogans* serovar Copenhageni strain FIOCRUZ L1-130 (LIC) was acquired from the Bacterial Zoonosis Laboratory at the Medicine Veterinary School at the University of São Paulo or from the UTHSC. LIC was originally isolated from a patient and donated from Fundação Instituto Oswaldo Cruz (FIOCRUZ), Salvador, Bahia, Brazil. LIC was previously sequenced (30).

LIC was propagated after passage in a hamster to maintain virulence. LIC was thawed from −80°C storage and cultured in Hornsby-Alt-Nally (HAN) semi-solid medium (31) at 29°C. For subsequent passages, 1 mL of the HAN culture was transferred into 9 mL of Difco *Leptospira* Medium Base EMJH (Ellinghausen–McCullough–Johnson–Harris, BD, ref. 279410) supplemented with Difco Enrichment EMJH (BD, ref. 279510). To preserve virulence, cultures were limited to two passages. Bacterial cells were harvested by centrifugation at 5,660 *× g* for 20 min at 20°C, resuspended in 10 mL sterile PBS, and washed twice under the same conditions. Leptospires were quantified using a Petroff-Hausser chamber under a dark-field microscope (ZEISS Scope. A1). A total of 10^8^ leptospires in 150 µL sterile endotoxin-free Dulbecco’s PBS (Lot # 3303187, Millipore Corp, Billerica, MA, USA) was injected intraperitoneally (i/p). Control mice received an equivalent volume of PBS and were kept under the same experimental conditions.

### Histopathological analysis

Kidney and liver samples were immediately placed in 4% formaldehyde and later transferred to 70% ethanol before processing. Tissue sections were fixed with Dubosq-Brasil solution (1 g picric acid; 150 mL of 80% ethanol; 60 mL of 40% formalin; and 15 mL of glacial acetic acid) for 2 h, post-fixed with 10% formalin (aqueous formaldehyde), and embedded in paraffin. Sections were stained with hematoxylin and eosin (HE) or immersed for 30 min in a 0.1% Sirius Red solution, followed by hematoxylin staining. Sirius Red staining highlights the presence of collagen type I and III fibers. A blinded histopathological analysis of the organs was performed by a pathologist (A.N.D.N.) from the Department of Pathology, Faculty of Medicine, University of São Paulo, São Paulo, Brazil, and an inflammatory score was assigned to the kidney and liver based on the following criteria: 0—no irregularities; 1—infiltrates detected, presence of apoptotic cells in <25% of the tissue. For kidney samples, nephritis and visible fibrosis were also noted; 2—infiltrates detected, with apoptotic cells in >25% but <50% of the tissue. For kidney samples, nephritis and visible fibrosis were observed. For liver samples, regeneration marks, duplicated nuclei, and hepatocyte destrabeculation were identified; 3—infiltrates detected, with apoptotic cells in >50% of the tissue. For kidney samples, nephritis and visible fibrosis were present. For liver samples, regeneration marks, duplicated nuclei, and hepatocyte destrabeculation were identified.

### Fibrosis quantification

Sirius Red-stained kidney sections were scanned with the ZEISS Axioscan 7 slide scanner. Images were analyzed in ZEISS ZEN BLUE 3.1 software. Red intensity was quantified using Image J software, with the green channel of the RGB stack command and a threshold of 0–110.

### qPCR and RT-PCR

The presence of LIC DNA in the liver and kidney was quantified by qPCR using a TAMRA-labeled molecular beacon probe (sequence: 5′-CTCACCAAGGCGACGATCGGTAGC-3′, FAM-TAMRA; Eurofins) and primers (sequences: F 5′-CCCGCGTCCGATTAG-3′ and R 5′-TCCATTGTGGCCGAACAC-3′; Eurofins) targeting leptospiral *16S rRNA*. DNA was extracted using the NucleoSpin Tissue Kit (#740952.250, Clontech, Mountain View, CA,USA), according to the manufacturer’s instructions. DNA concentration and purity were measured at 260/280 nm and 260/230 nm using a NanoDrop instrument (Thermo Scientific). qPCR was conducted alongside a standard curve of 10^6^ to 1 *L*. *interrogans* DNA copies.

Total cellular mRNA from the kidney tissue was extracted using RNeasy Mini Kit (#74104, Qiagen, Germany). RNA concentration and purity were assessed at 260/280 nm before proceeding with cDNA production. A high-capacity cDNA Reverse Transcriptase Kit (#4368814, Applied Biosystems) was used for cDNA preparation. TAMRA probes and primers for *COL1A1* (sequences: F 5′-TAAGGGTACCGCTGGAGAAC-3′, R 5′-GTTCACCTCTCTCACCAGCA-3′ and TAMRA probe 5′-AGAGCGAGGCCTTCCCGGAC-3′, FAM-TAMRA; Eurofins), *iNOS* (sequences: F 5′-GCTGGGCTGTACAAACCTTC-3′, R 5′-GCATTGGAAGTAGAAGCGTTTC -3′ and TAMRA probe 5′-GGCAGCCTGTGAGACCTTTGAT-3′, FAM-TAMRA; Eurofins), and *β-actin* (endogenous control, sequences: F 5′- CCACAGCTGAGAGGGAAATC-3′, R 5′-CCAATAGTGATGACCTGGCCG-3′ and TAMRA probe 5′- GGAGATGGCCACTGCCGCATC-3′, FAM-TAMRA; Eurofins) were used, along with Taqman Gene Expression Assays Kits for *FN1* and *ACTA1* (Mm01546133_m1 and Mm01256744_m1, Thermo Fisher Scientific).

The qPCR mixture contained a final mixture of 900 nM for each primer, 250 nM for the specific TAMRA probe, 10 µL of TaqMan Fast Advanced Master Mix (#4444557, Applied Biosystems), and 2 µL of the DNA or cDNA sample, for a volume of 20 µL. All reactions were performed in duplicate. The amplification was done in a QuantStudio 3 equipment, following the protocol: 2 min at 50°C, 20 s at 95°C, followed by 40 cycles of amplification (1 s at 95°C and 20 s at 60°C). Data for gene expression were analyzed with the ΔΔCt comparative method.

### Flow cytometry

Spleen, kidney, and six peripheral lymph nodes were collected from three mice per group. Cell suspensions were prepared, treated with red blood cells lysis solution using a previously described protocol with some modifications (32). Dead cells were enumerated and used to adjust the cell number using a live/dead cell stain and counted with a Luna Cell Counter (Logos Biosystems, South Korea). Viable cells (10^6^) were seeded per well in a 96-well microtiter plate and blocked with anti-mouse CD16/32 antibody (1:100) for 15–20 min on ice in PBS, pH 7.5. Cells were washed with cell staining buffer (Ca^2+^-free and Mg^2+^-free PBS containing 3% heat-inactivated fetal bovine serum [2R&D Systems, Bio-techne, MN, USA], 0.09% sodium azide [Sigma-Aldrich, St. Louis, MO, USA], 5 mM EDTA). Surface staining was performed with primary conjugated antibodies against various cell surface markers, incubated for 30 min at 4°C in the dark, and washed twice with cell staining buffer. Table S1 indicates the source of all the antibodies used for flow cytometry. Cells were fixed with 4% paraformaldehyde for 10 min, followed by a single PBS wash. Fixed cells were finally resuspended in cell staining buffer, and data analysis was performed using a BioRad ZE5 Cell. The data were analyzed using FlowJo software.

### Serum analysis of creatinine, uric acid, and urea

After euthanasia, mouse blood was collected through heart puncture and set to rest for 1 h in ice. The blood was centrifuged at 1,500 × *g*, and the serum was harvested and stored at −80°C until later use. Serum creatinine (# 27-500), uric acid (# 140-1/100), and urea (# 96-300) were analyzed with Labtest Kits (Minas Gerais, Brazil). Colorimetric tests were performed with 10 µL serum and compared to a standard solution (urea: 70 mg/dL; uric acid: 4 mg/dL; and creatinine: 4 mg/dL) provided by the manufacturer. Absorbance was measured using an Ultrospec 2100 Pro equipment (GE Healthcare, Chicago, USA). Urea, uric acid, and creatinine levels were determined at 600 nm, 505 nm, and 510 nm, respectively.

### Serum immunoglobulin analysis

The concentrations of serum specific anti-LIC IgM, IgG, and IgG subtypes (IgG1, IgG2b, and IgG3) were measured by ELISA. We utilized 10^6^ heat-killed LIC in 100 µL of carbonate buffer (Carbonate Coating Buffer CB01100, Thermo Scientific) to coat 96-well plates (MaxiSorp, Thermo Fisher Scientific, Waltham, MA, USA) overnight at 4°C. The plates were washed four times with 300 µL of PBS + 0.05% Tween (PBS-T) and incubated with 250 µL of blocking buffer (PBS-T + 1% fresh bovine serum albumin) for 1 h at 37°C. Followed by washing with PBS-T, the plates were dried and incubated for 1 h at 37°C with mouse serum (1/100) diluted in 100 µL of blocking buffer. After another washing with PBS-T, these plates were incubated with rabbit secondary horseradish peroxidase-conjugated antibodies, specific against mice IgM, IgG, IgG1, IgG2b, or IgG3 (1/10,000; Jackson ImmunoResearch, PA, USA). 3,3′,5,5′-tetramethylbenzidine (substrate solution N301, Thermo Scientific) and a stopping solution (N600, Thermo Scientific) were added to the wells, and optical density was determined with the SpectraMax Plus 384 ELISA reader spectrophotometer at 450 nM wavelength.

### Acute kidney injury with cisplatin injection

Cisplatin is a chemotherapeutic agent that induces acute kidney injury (33). To analyze the impact of *Leptospira* infection in mice with pre-existing renal injury, WT and C3KO mice were treated with a single (i/p) injection of cisplatin (10 mg/kg) (Millipore-SIGMA, #PHR1624) diluted in 150 µL of sterile PBS. Control groups received 150 µL of PBS alone. After 1 month, mice were further injected with 150 µL PBS (control) or with 10^8^ LIC in 150 µL (i/p). Animals were monitored daily for weight changes and clinical signs for 15 d.p.i. and then euthanized with isoflurane. Kidney, liver, and lung samples were collected for histopathological analysis, and part of them were treated with RNAprotect tissue reagent (Qiagen, ID: 76106) before DNA extraction.

### Proteome profile array

Mouse serum cytokines, including some chemokines, and Complement C5/C5a were detected using the Proteome Profiler Array Cytokine Mouse Kit (Panel A, catalog ARY006, R&D Systems, Minneapolis, MN, USA) (34, 35) following the manufacturer’s instructions. Array membranes were first treated with blocking buffer (provided by the manufacturer) for 1 h at room temperature in a rotary shaker. Approximately 100 µL of pooled serum per group was mixed for 1 h with a cocktail of 40 different biotinylated detection antibodies and then incubated overnight at 4°C with the Mouse Cytokine Array nitrocellulose membrane. The membranes contained spotted capture antibodies in duplicate and were washed to remove any non-binding conjugates. They were then incubated with a horseradish protease-conjugated anti-mouse IgG secondary antibody. Finally, the membranes were washed three times with wash buffer and developed using the developing solution provided in the kit. Chemiluminescence intensity was proportional to the amount of cytokine bound to the membrane. Images at different exposure times were captured using a Chemi-Doc image analyzer, and mean pixel density was quantified using QuickSpots software.

### Statistical analysis

Statistical analyses were performed using GraphPad Prism software or JASP (v. 0.19.1), and graphs were prepared with GraphPad on Windows 11. Prior to conducting ANOVA tests, groups were evaluated for normal population distribution using a Shapiro-Wilk test. Two-way ANOVA was performed, considering two independent factors: mouse genotype (WT or C3KO) and treatment type (control or LIC). When appropriate, *post hoc* multiple comparisons were conducted using Tukey’s correction, with a family-wise *α* of 0.05. If ANOVA was not applicable, the Kruskal-Wallis nonparametric test was used, followed by Dunn’s *post hoc* tests.

## RESULTS

### Survival and *Leptospira* renal colonization in infected mice

**Fig 1:**
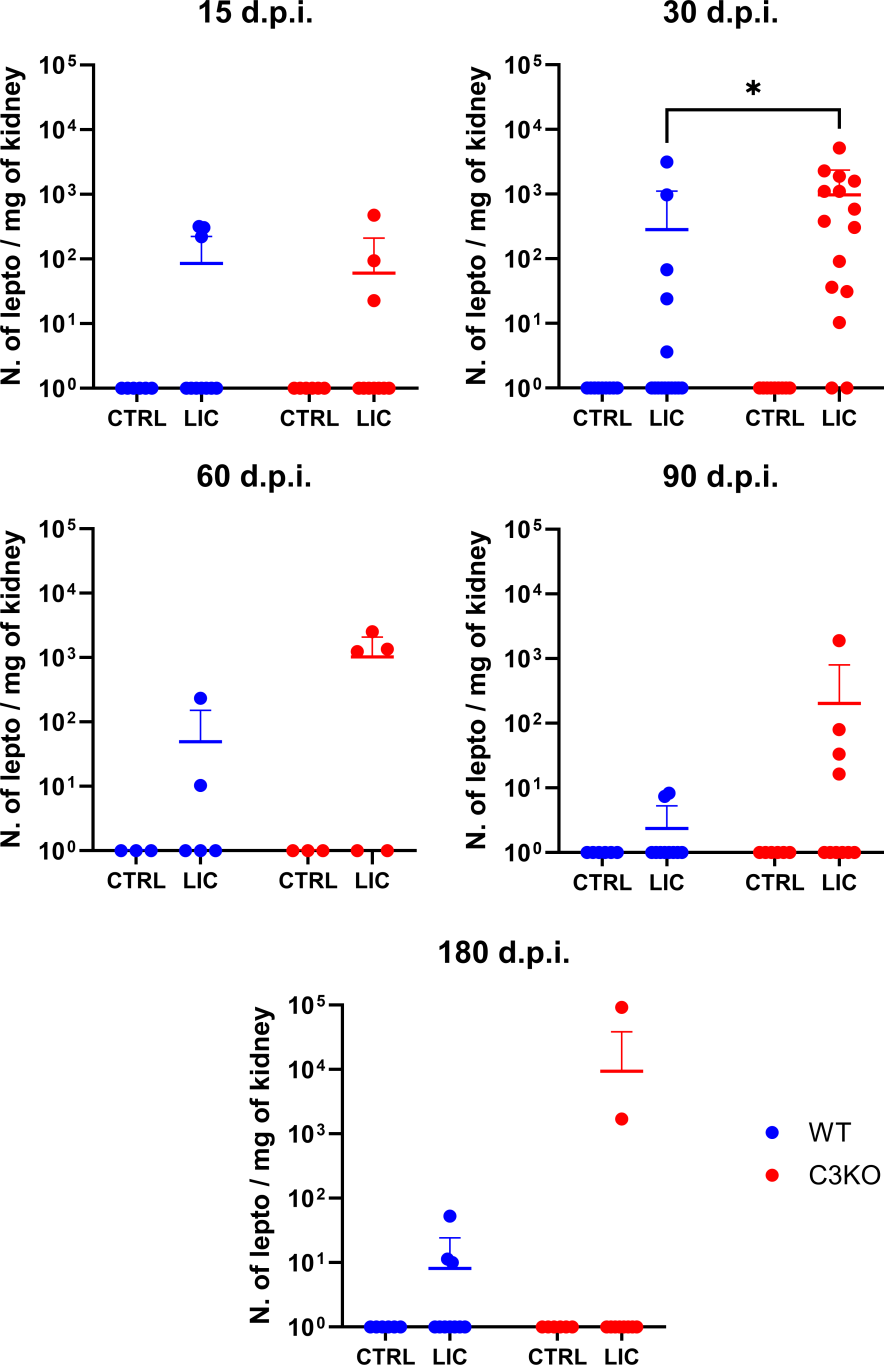
Quantification of kidney leptospiral load. Genomic DNA was extracted from the kidneys of WT and C3KO mice inoculated with PBS (control; CTRL) or 10^8^ *L. interrogans* serovar Copenhageni strain FIOCRUZ L1-130 (LIC) (i/p) and monitored for 15, 30, 60, 90, and 180 d.p.i. Leptospiral load was quantified using a standard qPCR curve and expressed as the number of leptospires (N. of lepto) per mg of kidney tissue. Statistical analysis was performed using Kruskal-Wallis nonparametric test, followed by Dunn’s *post hoc* test. Mice were obtained from the Animal Care Unit from ICB-USP. Infections at 15, 90, and 180 d.p.i. were repeated twice, while infection at 30 d.p.i. was performed thrice, and 60 d.p.i. was repeated once. Each dot represents one animal. *P < 0.05.

WT and C3KO mice were infected (i/p) with 10^8^ LIC or PBS (control) and monitored at 15, 30, 60, 90, and 180 d.p.i. All groups of mice survived, and body weight differences were not significant when comparing LIC-infected WT and C3KO mice even at 180 d.p.i. Splenomegaly was observed in infected mice at multiple time points when compared to uninfected controls. However, in the absence of C3, this increase was significantly attenuated at 30 d.p.i. compared to infected WT mice (Fig. S1). The presence of LIC in kidney tissue was quantified by qPCR (Fig. 1). A higher leptospiral load was observed in the kidneys of LIC-infected C3KO mice at 30 d.p.i. compared to WT counterparts.

### Renal injury during *Leptospira* infection

**Fig 2:**
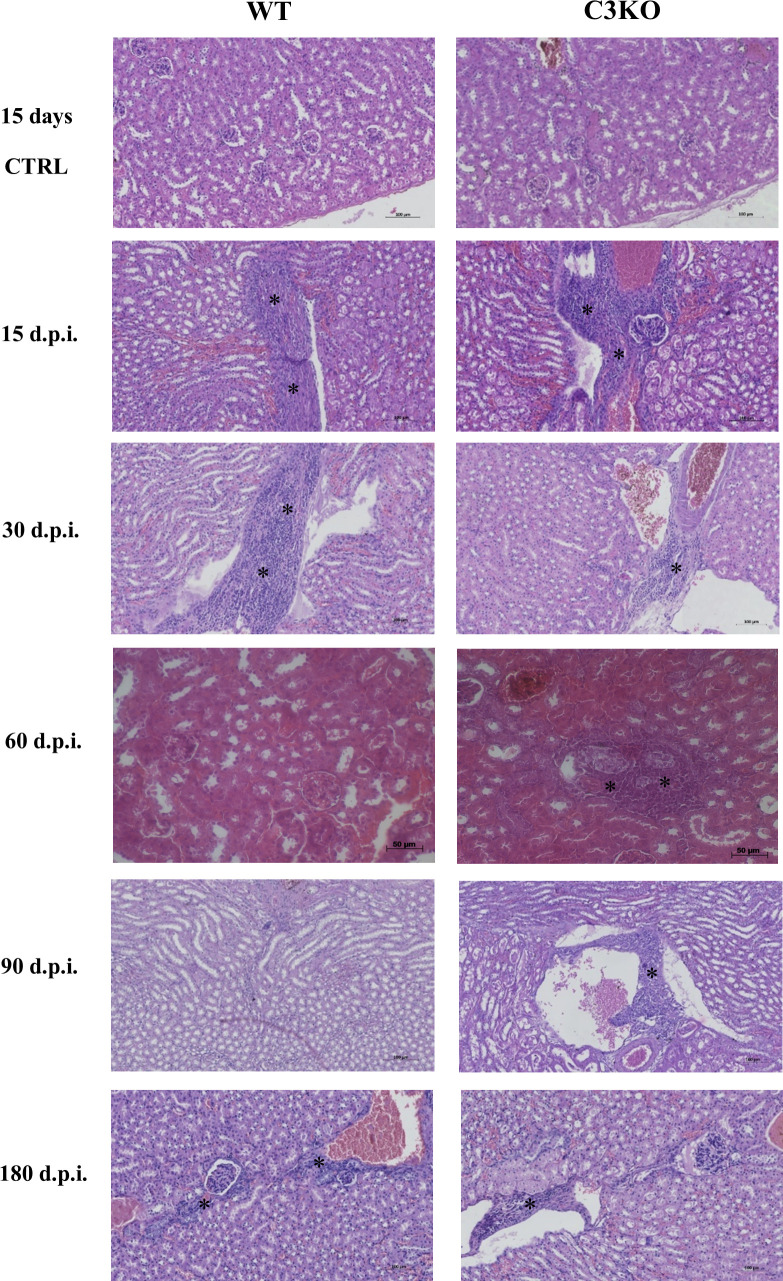
Kidney histopathological analysis after infection with pathogenic leptospires. WT and C3KO mice were inoculated with PBS (control; CTRL) or 10^8^ *L. interrogans* serovar Copenhageni strain FIOCRUZ L1-130 (LIC) (i/p) and monitored for 15, 30, 60, 90, and 180 d.p.i. Kidney sections were stained with HE. Inflammatory infiltrates (*), nephritis, and fibrosis were observed in both groups of LIC-infected mice. Images are shown at 200× magnification. Mice were obtained from the Animal Care Unit of ICB-USP.

**Fig 3:**
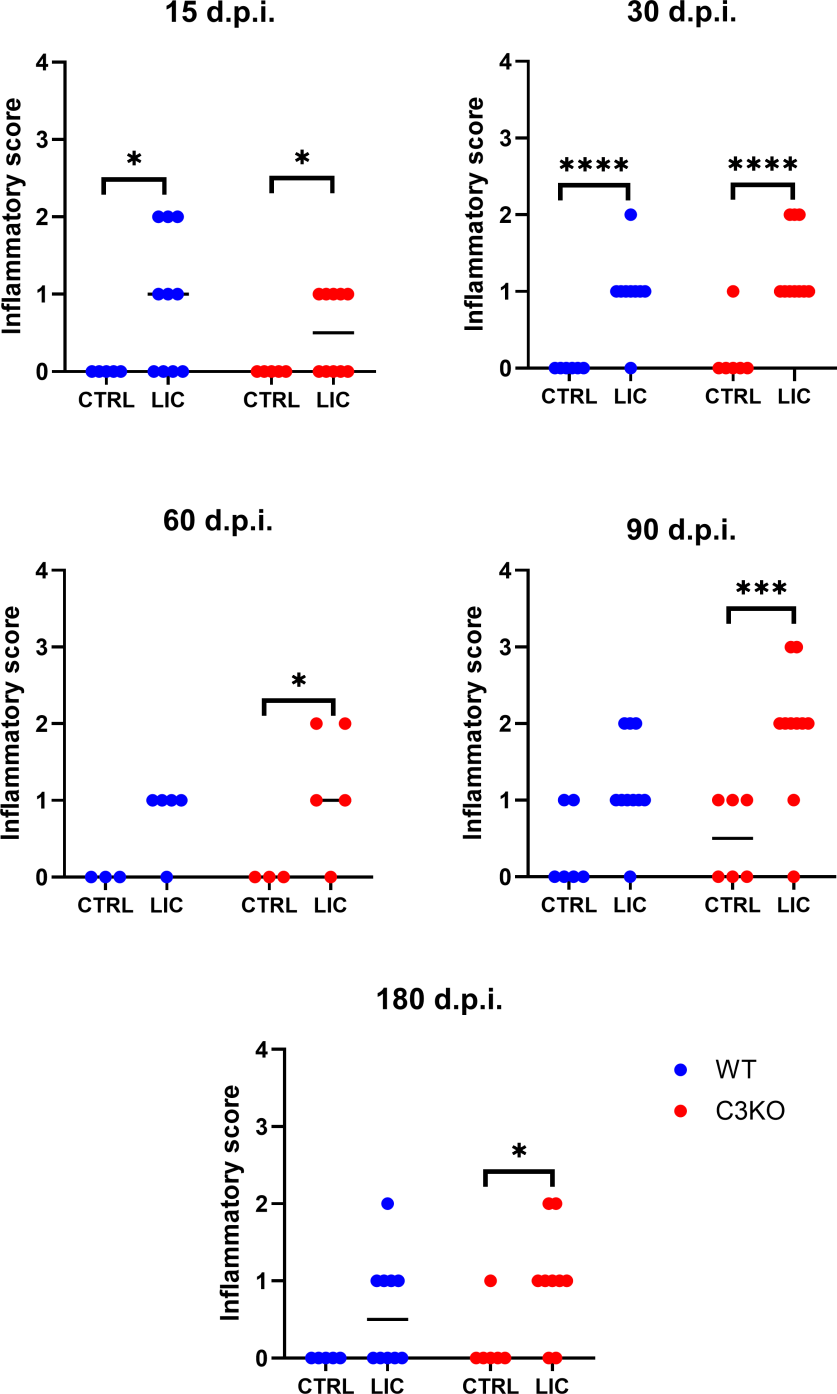
Renal inflammatory score. WT or C3KO mice were inoculated with PBS (control; CTRL) or 10^8^ *L. interrogans* serovar Copenhageni strain FIOCRUZ L1-130 (LIC) (i/p) and monitored for 15, 30, 60, 90, and 180 d.p.i. The score was classified as follows: 0, no alterations; 1, <25% of fibrosis, nephritis, and inflammatory infiltrates; 2, >25% and <50% of fibrosis, nephritis, and inflammatory infiltrates; and 3, >50% of fibrosis, nephritis, and inflammatory infiltrates. Statistical analysis was performed using the Kruskal-Wallis test (as the values are ordinal numbers), followed by Dunn’s *post hoc* test. *P < 0.05, ***P < 0.001, and ****P < 0.0001. Mice were obtained from the Animal Care Unit of ICB-USP. Infections at 15, 30, 90, and 180 d.p.i. were repeated twice, while infection at 60 d.p.i. was performed once. Each dot represents one animal.

Histopathological analysis of kidney samples (Fig. 2) showed that non-infected WT and C3KO mice were apparently healthy, with no inflammatory cell infiltration. In contrast, kidneys from both groups of LIC-infected mice at 15 and 30 d.p.i. exhibited inflammatory cell infiltrates near the vascular endothelium, fibrosis, and nephritis. Inflammation was observed in the kidney of infected WT mice after 60, 90, and 180 d.p.i. However, this occurrence was not statistically significant when compared to non-infected WT mice. Although no significant differences were observed between LIC-infected WT and C3KO mice, C3KO mice displayed persistent kidney injury even after 180 d.p.i. when compared to non-infected C3KO control (Fig. 3). On the other hand, LIC-infected WT mice showed no significant tissue damage after 2, 3, or 6 months of infection compared to non-infected WT mice.

We also investigated if kidney lesions caused by LIC would be exacerbated by an existing kidney lesion (in this case caused by cisplatin, a known nephrotoxic chemotherapeutic drug) (33). While C3 seemed to play a role in either augmenting or diminishing the lesions, the injury caused by both was higher compared to mice only infected with LIC or only treated with cisplatin (Fig. S2).

### Parameters of renal function and fibrosis formation during *Leptospira* infection

**Fig 4:**
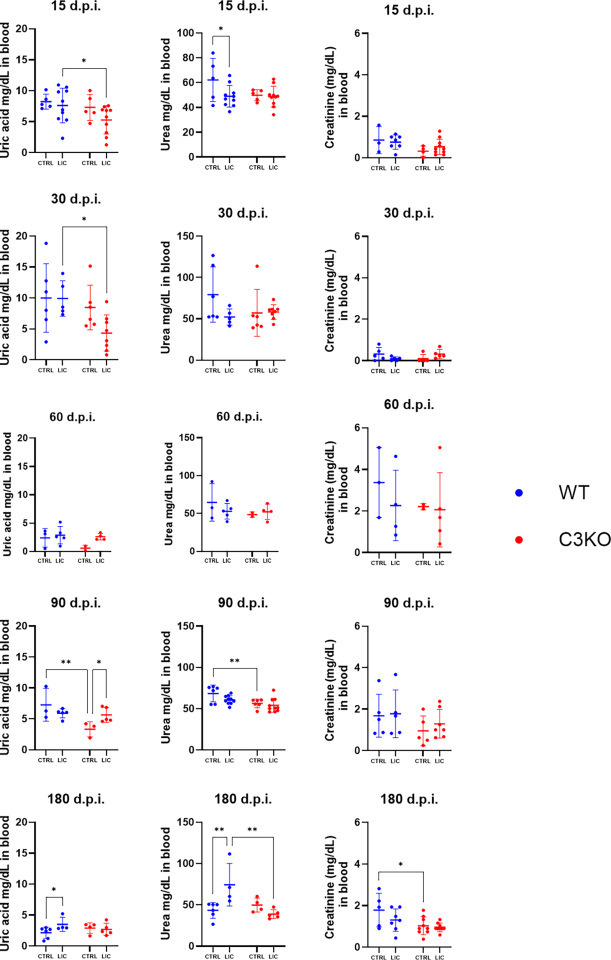
Serum levels of uric acid, urea, and creatinine. WT or C3KO mice were inoculated with PBS (control; CTRL) or with 10^8^ *L. interrogans* serovar Copenhageni strain FIOCRUZ L1-130 (LIC) (i/p). Data are represented as mean ± S.D. Each dot represents one animal. Statistical analysis was performed using two-way ANOVA, followed by Tukey’s test, with familiar *α* of 0.95. *P < 0.05 and **P < 0.01. Mice were obtained from the Animal Care Unit of ICB-USP. Infections at 15, 30, 90, and 180 d.p.i. were repeated twice, while infection at 60 d.p.i. was performed once. Each dot represents one animal.

Renal function was assessed by measuring serum levels of urea, uric acid, and creatinine (Fig. 4) in both infected and non-infected WT and C3KO mice. The lack of C3 influenced uric acid levels, leading to a reduction in serum levels at 15 and 30 d.p.i. and subsequently resulting in a decrease in serum urea levels at 180 d.p.i., with significant differences observed when compared to WT-infected mice. In contrast, serum creatinine levels remained similar between WT and C3KO-infected animals.

**Fig 5:**
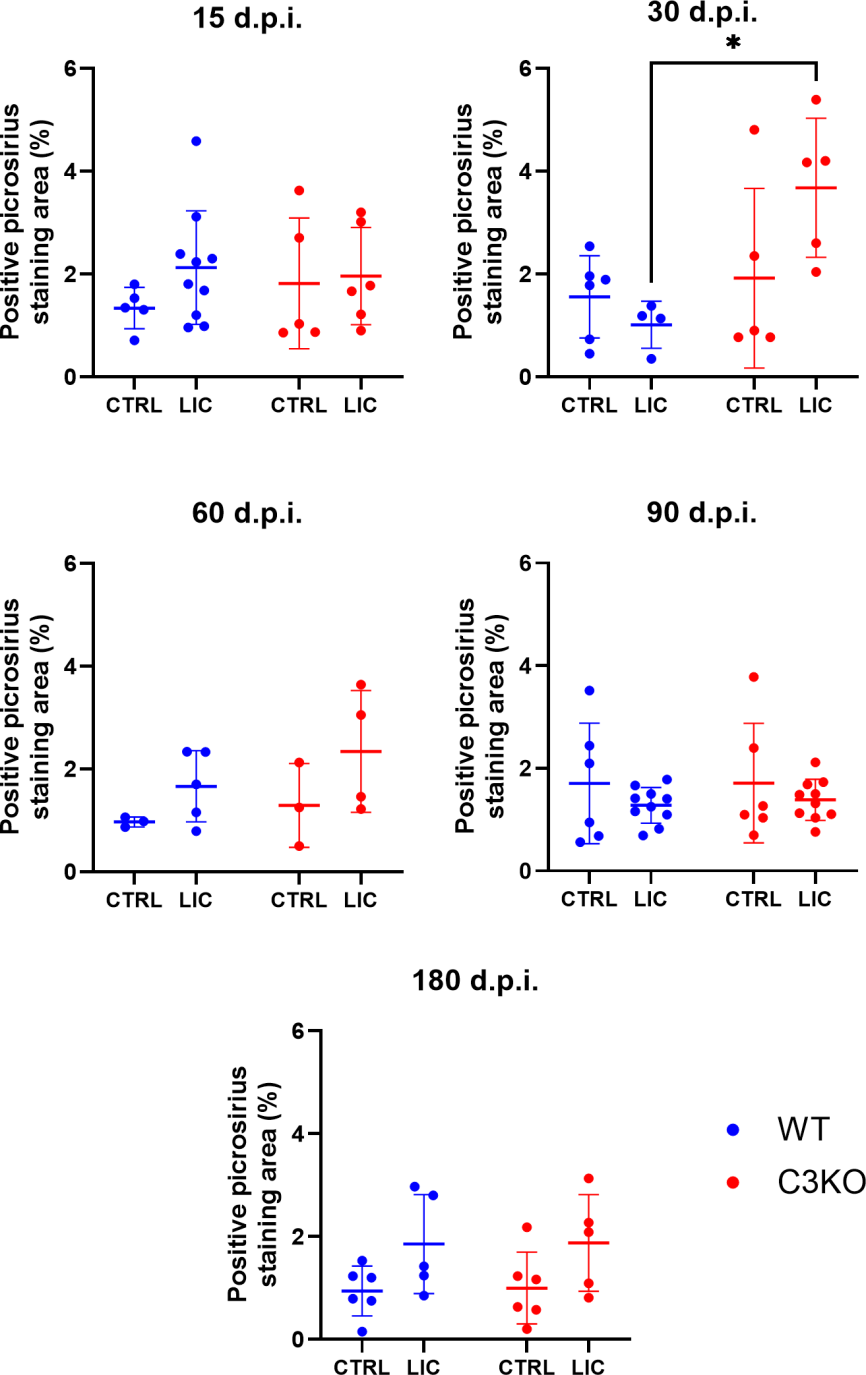
Fibrosis area percentage during LIC infection. WT or C3KO mice were FIOCRUZ L1-130 (LIC) (i/p) and followed from 15 d.p.i. up to 6 months (180 d.p.i.). Sirius Red-stained kidney sections were scanned with the ZEISS Axioscan 7 slide scanner. Red intensity was quantified using Image J. Statistical analysis was performed using two-way ANOVA, followed by Tukey’s test. *P < 0.05. Mice were obtained from the Animal Care Unit of ICB-USP. Infections at 15, 30, 90, and 180 d.p.i. were repeated twice, while infection at 60 d.p.i. was performed once. Each dot represents one animal.

Additionally, fibrosis formation in LIC-infected mice was monitored (Fig. S3). The absence of C3 was significantly associated with larger areas of kidney fibrosis at 30 d.p.i. compared to WT mice (Fig. 5). The time point 30 d.p.i. seems to be critical in this murine model for demonstrating the role of C3 during *Leptospira* infection. At this point, C3-deficient mice carried a higher leptospiral burden in the kidneys (Fig. 1), accompanied by increased fibrosis compared to WT counterparts. This suggests that the delayed clearance of leptospires in the absence of C3 provides prolonged stimulation, thereby promoting renal fibrosis.

We also analyzed the mRNA production of fibrosis-related genes, including fibronectin (*FN1*), collagen-1 (*COL1A1*), and alpha-smooth muscle actin (*α-SMA*) in the kidney. However, except for *α-SMA* at 15 d.p.i., neither C3 deficiency nor LIC infection appeared to significantly alter these parameters (Fig. S4).

### Production of specific anti-LIC antibodies

**Fig 6:**
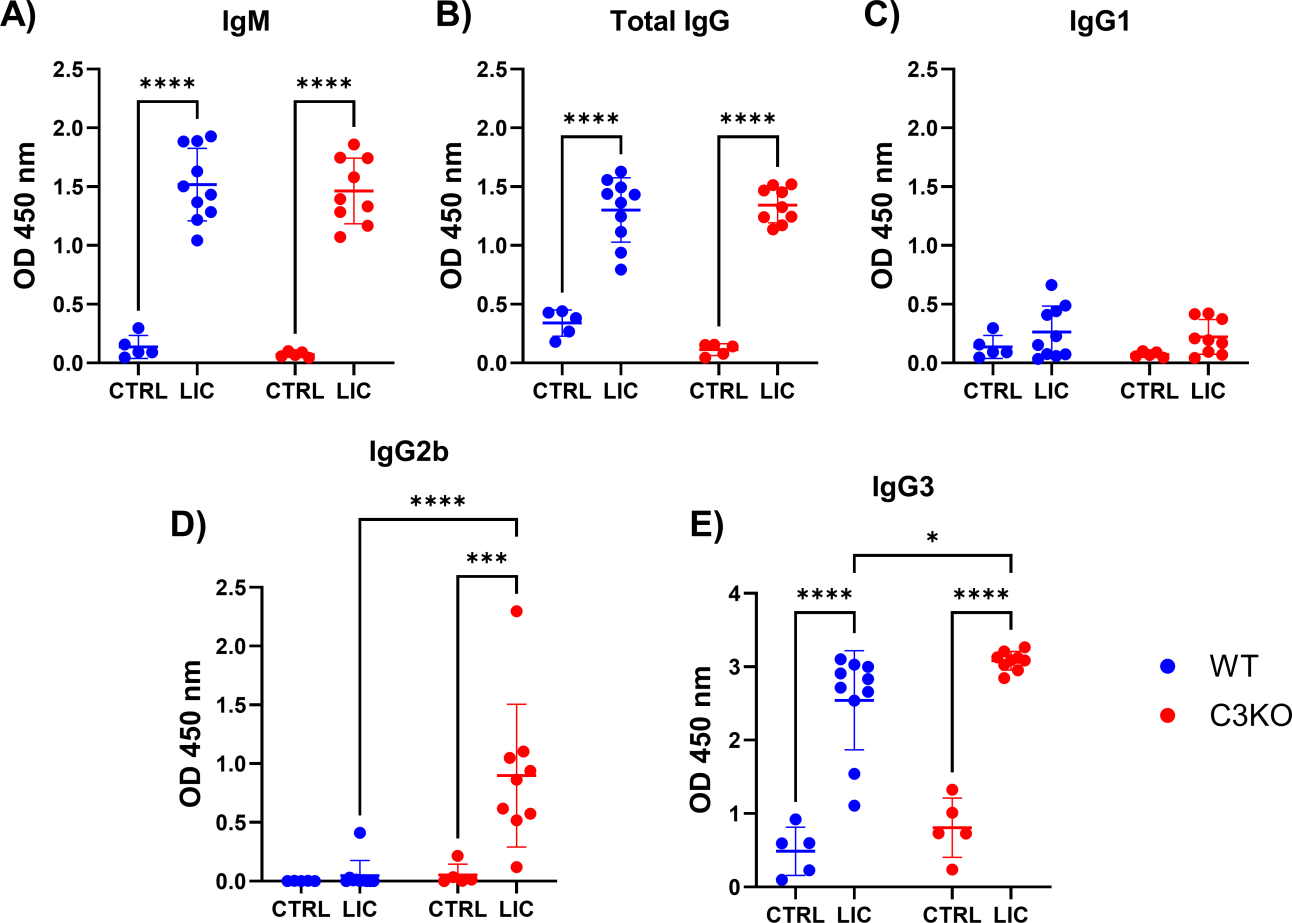
Quantification of specific antibodies against LIC. WT or C3KO mice were inoculated with PBS (control, CTRL) or 10^8^ *L. interrogans* serovar Copenhageni strain FIOCRUZ L1-130 (LIC) (i/p). After 15 d.p.i., serum was obtained, and levels of specific IgM, total IgG, and other IgG subclasses anti-LIC were quantified by ELISA. Wells were coated with 10^6^ heat-killed LIC and incubated with diluted mice serum (1:100). Secondary antibodies against (**A**) IgM, (**B**) total IgG, (**C**) IgG1, (**D**) IgG2b, and (**E**) IgG3 were used (1:5,000). Each dot represents one animal (*n* = 5–6 for PBS groups; *n* = 5–10 for infected groups). Statistical analysis was performed using two-way ANOVA followed by Tukey’s test, with familiar *α* of 0.95. *P < 0.05, ***P < 0.001, and ****P < 0.0001. Mice were obtained from the Animal Care Unit of ICB-USP. CTRL group (*n* = 5) and LIC-infected group (*n* = 9-10). Data represent two independent experiments.

Both groups of mice were infected with viable LIC, and the production of specific anti-LIC antibodies was assessed in serum at 15 d.p.i. (Fig. 6) and 30 d.p.i. (Fig. S5). Both WT and C3KO LIC-infected mice exhibited significantly higher serum levels of IgM (Fig. 6) compared to non-infected control mice. No significant differences were observed in the serum of specific IgM, total IgG, and IgG1 between WT and C3KO LIC-infected mice. However, in the absence of C3, LIC-infected mice produced higher levels of IgG2b (Fig. 6D) and IgG3 (Fig. 6E) compared to WT counterparts, likely due to prolonged leptospiral survival in these deficient animals. Similar results were observed at 30 d.p.i. (Fig. S5), except for lower IgM levels compared to the 15 d.p.i. group.

### Effect of C3 on effector T cell populations during *Leptospira* infection

**Fig 7:**
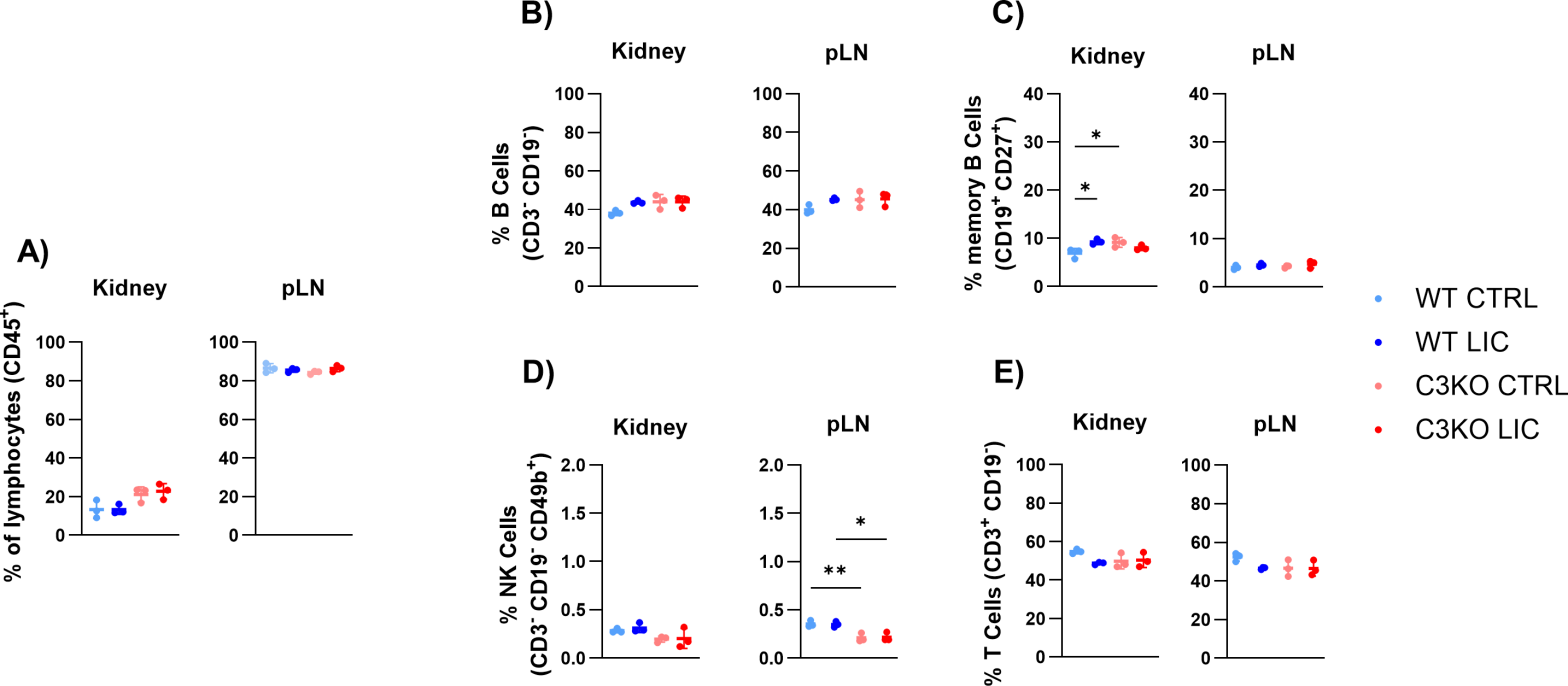
Immune cell populations in the kidney and peripheral lymph nodes (pLN). WT or C3KO mice were inoculated with PBS (control; CTRL) or with 10^8^ *L. interrogans* serovar Copenhageni strain FIOCRUZ L1-130 (LIC) (i/p). Graphs represent the percentage of CD45^+^ T and B lymphocytes (**A**), the total of B cells (**B**), memory B cells (**C**), NK cells (**D**) and T cells (**E**) after 30 days post-infection. Each dot represents one animal (*n* = 3 per group). Statistical analysis was performed using two-way ANOVA, followed by Tukey’s test, with familiar *α* of 0.95. *P < 0.05, **P < 0.01, and ns: non-significant. Mice were obtained from the Animal Care Unit of UTHSC.

Flow cytometry analysis of immune cells in the kidney, lymph nodes, and spleen revealed no significant differences in the percentage of lymphoid cells (CD45^+^), B lymphocytes (CD19^+^), and T (CD3^+^) lymphocytes among the four experimental groups (non-infected vs LIC-infected; WT vs C3KO) (Fig. 7; Fig. S6).

**Fig 8:**
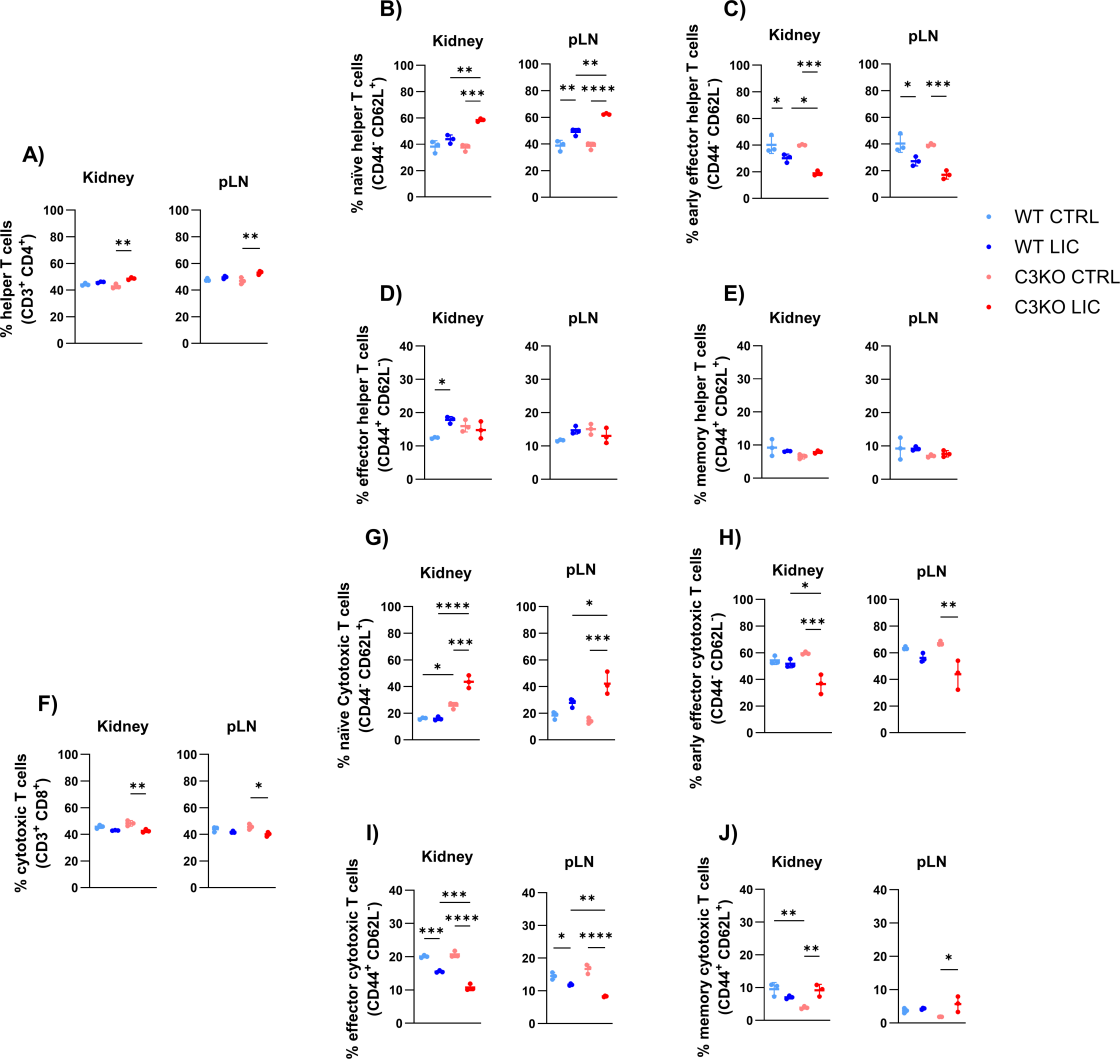
Helper T (CD4^+^) and cytotoxic T (CD8) cell populations in the kidney and peripheral lymph nodes (pLN). WT or C3KO mice were inoculated with PBS (control; CTRL) or with 10^8^ *L. interrogans* serovar Copenhageni strain FIOCRUZ L1-130 (LIC) (i/p). Graphs represent the percentage of CD4^+^ and CD8^+^ T cells (**A and F**) and their subpopulations of helper T cells (**B and G**), early effector helper T cells (**C and H**), effector helper T cells (**D and I**) and memory helper T cells (**E and J**) at 30 days post-infection. Each dot represents one animal (*n* = 3 per group). Statistical analysis was performed using two-way ANOVA, followed by Tukey’s test, with familiar *α* of 0.95. *P < 0.05, **P < 0.01, ***P < 0.001, ****P < 0.0001, and ns: non-significant. Mice were obtained from the Animal Care Unit of UTHSC.

However, in the absence of C3, a lower proportion of effector T cells was detected in both the kidney and lymph node, but not in the spleen, of infected mice, suggesting a delay in the differentiation of naïve T cells into effector T cytotoxic cells (Fig. 8). A similar trend was observed in early effector T helper lymphocytes, indicating that C3 may play a crucial role in promoting local T cell differentiation (Fig. 8B and C). Additionally, NK cells were present in proportionately low numbers in the lymph nodes of C3KO mice; however, this difference was relatively minor compared to WT counterparts (Fig. 7D).

### Cytokine production

A proteome profile array was used to analyze the presence of 40 cytokines (including chemokines) and Complement C5/C5a protein in pooled serum (Fig. S7). At 30 d.p.i., most cytokines were below the detectable threshold (3%), and the detectable ones were lower in the infected group than their respective controls. Similarly, chemokine levels were generally reduced in infected groups compared to controls, except for IP-10 and KC in WT mice and sICAM-1 in C3KO mice, which remained unchanged. The relative level of C5/C5a was higher in the WT uninfected group compared to the LIC-infected counterpart, but this difference was not detected in the C3KO groups.

## DISCUSSION

Rodents represent the primary reservoirs and transmitters of *Leptospira* in both natural and urban environments. Their ability to sustain persistent renal colonization is likely due to continuous exposure to the microorganism, coupled with their natural resistance and asymptomatic carriage. Nevertheless, certain mouse strains, such as C3H/HeJ (36) and TLR4/TLR-2 double knockout mice (37), have proven to be valuable experimental models for leptospirosis research. In contrast, humans are considered accidental hosts. While most infected individuals remain asymptomatic, approximately 5%–10% develop severe disease (1), although the mechanisms underlying these differences between human and murine infection remain unclear. Importantly, both human and murine sera can kill non-pathogenic *Leptospira* spp. via the Alternative Pathway, whereas pathogenic strains exhibit marked resistance to Complement-mediated killing (22). This ability to subvert Complement attack highlights the importance of Complement components, particularly C3, in host defense. Understanding the role of C3 during infection may therefore provide critical insight into the balance between resistance, asymptomatic carriage, and progression to severe disease.

This study investigates the role of murine C3 during tissue colonization of pathogenic *L. interrogans* and its potential contribution to kidney fibrosis during chronic infection. Establishing a model for live, chronic leptospiral infection is a complex but critical step toward understanding the mechanisms by which leptospirosis contributes to chronic kidney disease. Here, we provide the first report of chronic infection using C57BL/6J mice and their C3 knockout counterparts, shedding light on C3’s role in the immune response to *Leptospira*.

The absence of C3 was not enough to cause more injury in LIC-infected mice even at 180 d.p.i. compared to their WT counterparts. This resilience is likely due to compensation by other immune mechanisms. However, kidney colonization by *L. interrogans* was higher in LIC-infected C3KO mice at 30 d.p.i., where 9 out of 15 mice displayed leptospiral loads exceeding 10^2^ leptospires/mg tissue, compared to minimal colonization in WT mice (only 2 in 15 mice) (Fig. 1). These findings suggest that while the absence of C3 does not influence disease severity, it does increase kidney colonization by *L. interrogans* in C57BL/6J mice at 30 d.p.i. Recently, our group demonstrated that during the early stages of infection with *L. interrogans* serovar Kennewicki strain Pomona Fromm, leptospiral loads were significantly higher, in the absence of C3, in the kidney, liver, and spleen at 3 d.p.i., and in the urine at 6 d.p.i. compared to WT-infected mice (22). These observations indicate that C3 plays a critical role in controlling bacterial load and kidney colonization during the initial phase of infection, and its influence persists even after 1 month after infection.

Kidney fibrosis has recently been associated with prolonged leptospirosis, characterized by extracellular matrix collagen deposition, nephritis, and sustained inflammation (38–40). However, the mechanisms driving the progression from the acute to the chronic stage of leptospirosis, and if C3 plays a role in this process, remain unknown. Here, although larger fibrotic areas were observed in C3KO mice at 30 d.p.i., the expression of fibrosis-related genes (e.g., *FN1*, *COL1A1*, and *α-SMA*) did not differ significantly between groups. These results contrast with findings from other murine models with genetic modification, such as *iNOS* KO mice (41), where FN1 and α-SMA levels were lower after LIC infection, as well as DAF-1 KO mice (42), and humanized TLR4 mice (43), which demonstrated a higher progression of fibrosis, evidenced by increased COL1A1 expression at different time points (90 d.p.i. for DAF-1 KO mice and 15 d.p.i. for humanized TLR4 mice [44]). Our findings suggest that C3 does not significantly influence the expression of fibrosis-related genes during LIC infection, suggesting that other pathways may play a more dominant role in fibrosis development.

C3-derived fragment C3d plays a pivotal role in immune modulation. C3d-bound antigens interact with Complement Receptor 2 (CR2, also known as CD21) on B lymphocytes, promoting their activation, memory formation, and immunoglobulin class switching (45). Vassalakis et al. (22) demonstrated that C3KO mice immunized with 3 × 10^7^ *L. interrogans* serovar Kennewicki strain Pomona Fromm or with 5 µg of recombinant leptospiral protein LigBC produced significantly lower levels of specific IgG compared to their WT counterparts. In that study, the same animals received a booster dose 2 weeks later, further highlighting reduced IgG production in the absence of C3.

Based on these findings, we expected that C3KO mice infected with a single dose of 10^8^ LIC would also exhibit lower levels of specific antibodies compared to WT LIC-infected mice. However, specific antibody levels remained similar or, in some cases, even higher despite the absence of C3. One possible explanation is that C3 deficiency allows pathogenic leptospires to persist for a longer period, leading to increased bacterial multiplication and, consequently, a higher antigen load. Another possibility is that the higher infectious dose used in this study may have masked differences in antibody production, as such differences are typically more pronounced at lower antigen doses (45) or during secondary immune responses (46).

Interestingly, after 30 d.p.i., the IgG response was predominantly composed of IgG3. In mice, IgG3 is associated with T cell-independent responses against lipopolysaccharide (LPS) antigens (47–49), whereas in humans, this response is driven by IgG2 (50, 51). This suggests that the absence of C3d may influence the T cell-independent response against LPS. Vernel-Pauillac and collaborators (52) also reported the production of specific IgM, IgG1, IgG2b, IgG2c, and IgG3 in C57/Black 6 mice infected with three different strains of pathogenic *Leptospira* even after 180 days of infection. Immunoglobulins play important roles, such as in the opsonization (52) and phagocytosis (53, 54) of *Leptospira*. However, pathogenic strains can cleave Fc receptors (29) and thereby evade phagocytosis. Whether different IgG subtypes have different roles in the immune response against *Leptospira* remains unknown.

Contrary to expectations, chronic LIC infection had only a minor impact on renal functional serum parameters. These findings are consistent with findings in galectin-3 knockout mice (55), where no significant deviations in renal biomarkers were observed. Such deviations may be unique to human leptospirosis (56, 57), as murine models appear to be more resilient to these changes. In leptospirosis patients, hypokalemia is commonly observed due to excessive urinary potassium excretion (58, 59). Measuring potassium levels in murine models of leptospirosis could provide valuable insights and should be considered in future studies.

The cellular immune response to pathogenic *Leptospira* also remains poorly understood. Here, we analyzed the lymphocyte populations after 30 d.p.i. In the absence of C3, *L. interrogans* infection led to a relatively minor reduction in the number of cytotoxic T CD8^+^ lymphocytes in the kidney and lymph nodes but not in the spleen, which may contribute to chronic local *Leptospira* infection. Pathogenic *L. interrogans* is known to induce cell death processes such as necroptosis in different immune cells (60), a process that exacerbates persistent inflammation during the early stages of infection. Blocking the necroptosis pathway has been shown to attenuate the effects of acute leptospirosis (61). This necroptosis mechanism may account for the lower abundance of effector cytotoxic T CD8^+^ lymphocytes during chronic infection, allowing leptospires to persist in the kidney, which serves as the primary site of inflammation during leptospirosis (62). Further studies are needed to fully elucidate this phenomenon.

Additionally, LIC-infected C3KO mice exhibited a higher proportion of naïve T lymphocytes and a lower proportion of early effector CD4^+^ and CD8^+^ T lymphocytes in the kidney and peripheral lymph nodes. C3 is produced locally during interactions between antigen-presenting cells and T lymphocytes, where it provides costimulatory signals and maintains the viability of naïve T cells (62–64). It also supports the activation and expansion of CD4^+^ and CD8^+^ T lymphocytes in response to viral (65, 66) and bacterial, such as *Listeria monocytogenes* (67), infections. Moreover, the local production of C3 appears critical for differentiation of naïve T lymphocytes into effector cells; in its absence, C3KO mice retain a relatively elevated proportion of naïve T cells, underscoring the importance of C3 in promoting the differentiation and expansion of effector T cell populations necessary to combat infection.

Overall, the absence of C3 does not impact mouse survival during leptospirosis. However, 1-month post-infection appears to be a critical time point in murine leptospirosis if C3 deficiency is present, as both leptospiral load in the kidney and fibrosis formation were more pronounced at this stage. Additionally, murine C3 deficiency impairs the differentiation of naïve T cells into effector helper and cytotoxic T lymphocytes in the kidney and lymph nodes after 30 d.p.i., suggesting that the depletion of cytotoxic T lymphocytes in the absence of C3 may contribute to chronic leptospirosis. Our findings highlight the potential role of C3 in regulating effector helper and cytotoxic T lymphocytes, serving as a key link between the innate and adaptive immune system in leptospirosis.

## SUPPLEMENTAL MATERIAL

Figure S1 iai.00398-25-s0001.docx Spleen weight to final weight ratio.

Figure S2 iai.00398-25-s0002.docx Kidney inflammatory score with cisplatin treatment.

Figure S3 iai.00398-25-s0003.docx Example of Red Sirius staining of kidney for data collection.

Figure S4 iai.00398-25-s0004.docx Fibrosis genes analyzed.

Figure S5 iai.00398-25-s0005.docx ELISA for immunoglobulin subtypes after 30 d.p.i.

Figure S6 iai.00398-25-s0006.docx Spleen cytometry analysis.

Figure S7 iai.00398-25-s0007.docx Proteome analysis of the serum.

Table S1 iai.00398-25-s0008.docx Antibody panel for cell cytometry.

ASM does not own the copyrights to Supplemental Material that may be linked to, or accessed through, an article. The authors have granted ASM a non-exclusive, world-wide license to publish the Supplemental Material files. Please contact the corresponding author directly for reuse.
