# Supplementary material for: Complement C3 deficiency enhances renal leptospiral load and inflammation while impairing T cell differentiation during chronic Leptospira interrogans infection
Source: Infect Immun. 2025 Nov 18;93(12):e00398-25. doi: 10.1128/iai.00398-25 (PMC12707143; doi:10.1128/iai.00398-25)
Supplement: Figure S1 — Spleen weight to final weight ratio. [file iai.00398-25-s0001.docx]

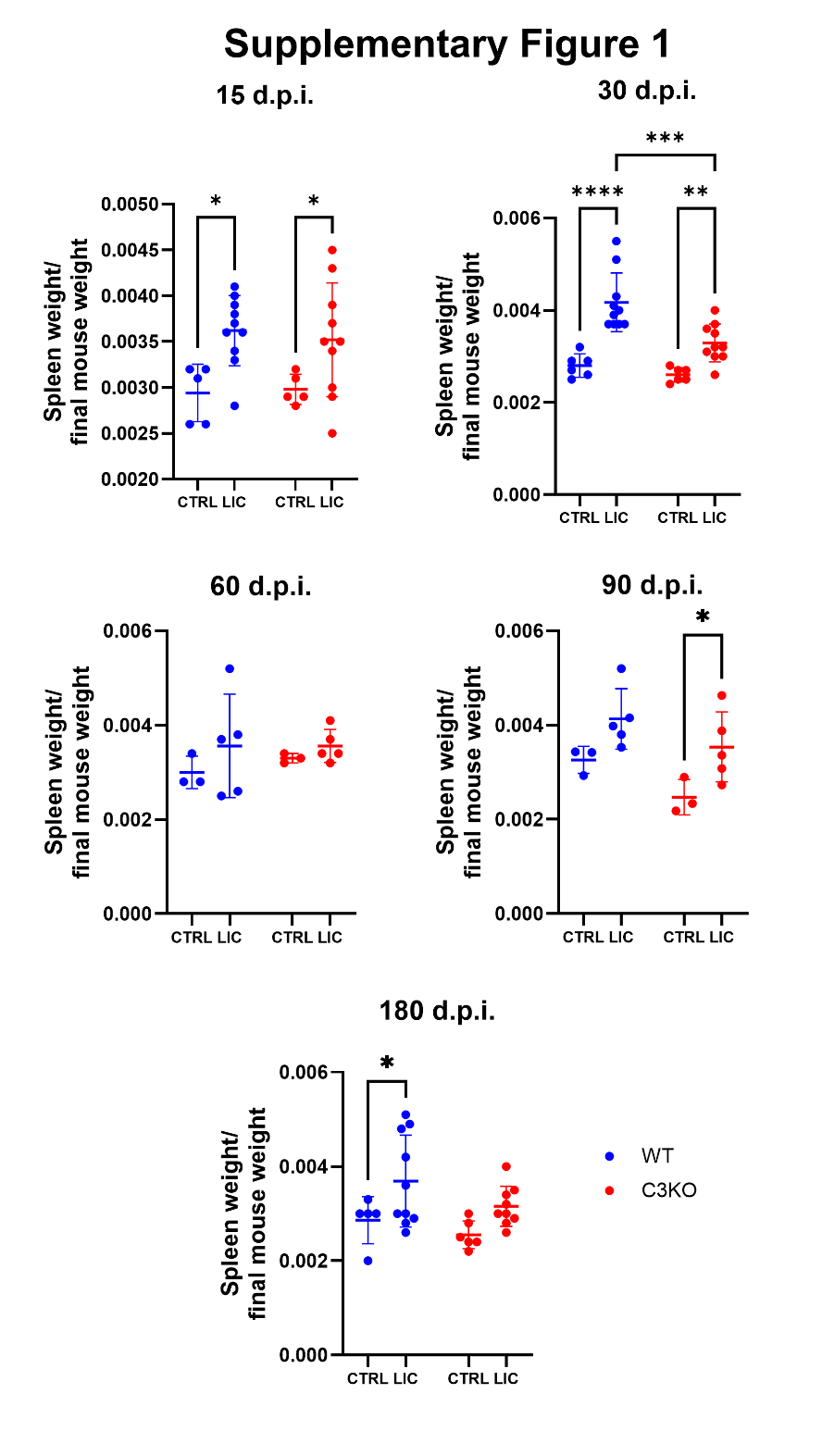


**Supplementary Fig 1. Spleen-to-total body weight ratio.** The spleen weight of WT and C3KO mice, injected with PBS (control) or 10^8^ *L interrogans* serovar Copenhageni strain FIOCRUZ L1-130 (LIC) (i/p) was measured after euthanasia and compared to the respective total body weight. Each dot represents one animal. Before statistical analysis, variance homogeneity of and normality of the groups was verified. A two-way ANOVA was performed, followed by Tukey’s post-hoc test. Significance was set at α = 0.05. **p* < 0,05; ***p* < 0,01; ****p* < 0,005, and *****p* < 0,001. Mice were obtained from the Animal Care Unit from ICB-USP. Infections at 15, 30, 90 and 180 d.p.i. were repeated twice, while infection at 60 d.p.i. was performed once.
