# Supplementary material for: Complement C3 deficiency enhances renal leptospiral load and inflammation while impairing T cell differentiation during chronic Leptospira interrogans infection
Source: Infect Immun. 2025 Nov 18;93(12):e00398-25. doi: 10.1128/iai.00398-25 (PMC12707143; doi:10.1128/iai.00398-25)
Supplement: Figure S2 — Kidney inflammatory score with cisplatin treatment. [file iai.00398-25-s0002.docx]

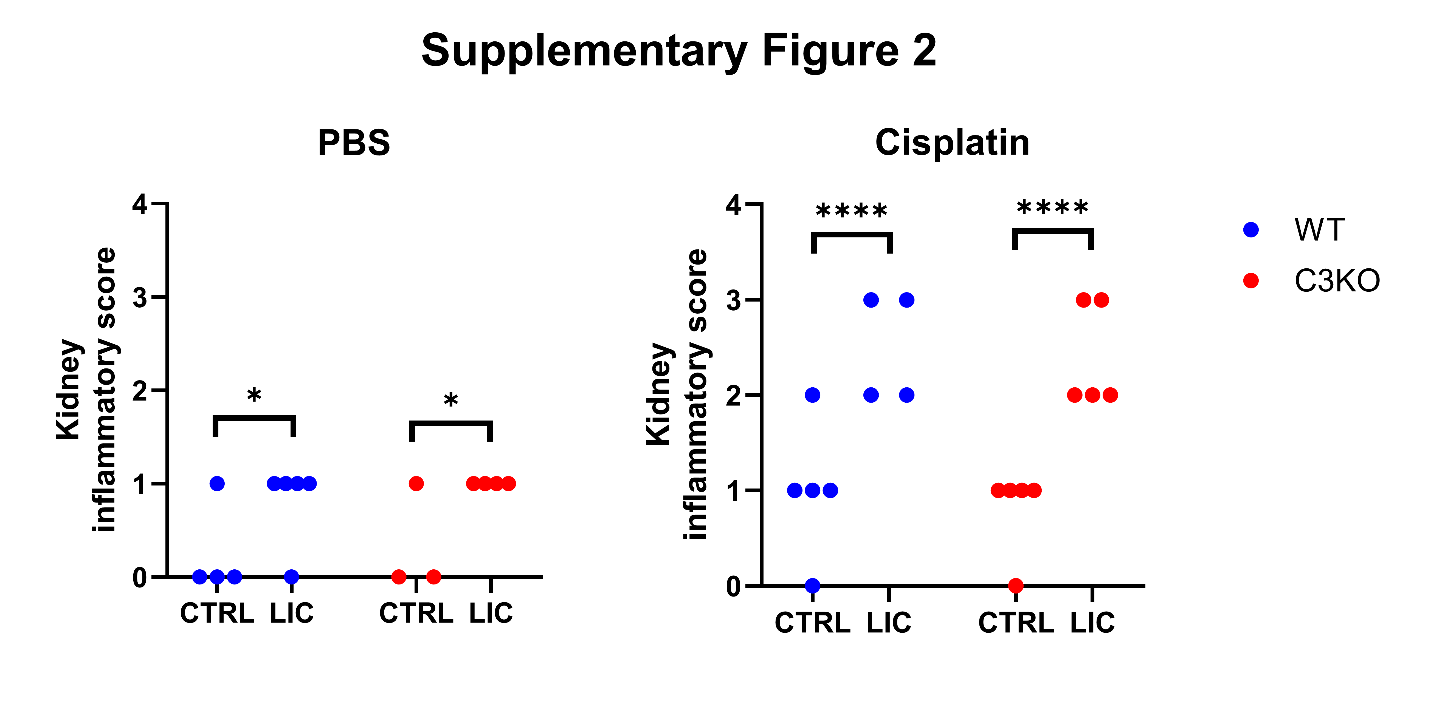


**Supplementary Fig 2. Kidney inflammatory score after cisplatin treatment.** WT and C3KO mice injected with PBS (CTRL) or cisplatin. Thirty days later, the mice were subsequently inoculated with PBS (control; CTRL) or 10^8^ *L interrogans* serovar Copenhageni strain FIOCRUZ L1-130 (LIC) (i/p). The inflammatory score was classified as follows: 0 – no alterations; 1 - < 25% of fibrosis, nephritis, and inflammatory infiltrates; 2 – 25% < and < 50% of fibrosis, nephritis, and inflammatory infiltrates; and 3 - 50% < % of fibrosis, nephritis, and inflammatory infiltrates. Statistical analysis was performed using Kruskal-Wallis. **p* < 0.05; and *****p* < 0.001. Mice were obtained from the Animal Care Unit from ICB-USP. Mice were obtained from the Animal Care Unit from ICB-USP. Each group n=5.
