# Supplementary material for: Complement C3 deficiency enhances renal leptospiral load and inflammation while impairing T cell differentiation during chronic Leptospira interrogans infection
Source: Infect Immun. 2025 Nov 18;93(12):e00398-25. doi: 10.1128/iai.00398-25 (PMC12707143; doi:10.1128/iai.00398-25)
Supplement: Figure S3 — Example of Red Sirius staining of kidney for data collection. [file iai.00398-25-s0003.docx]

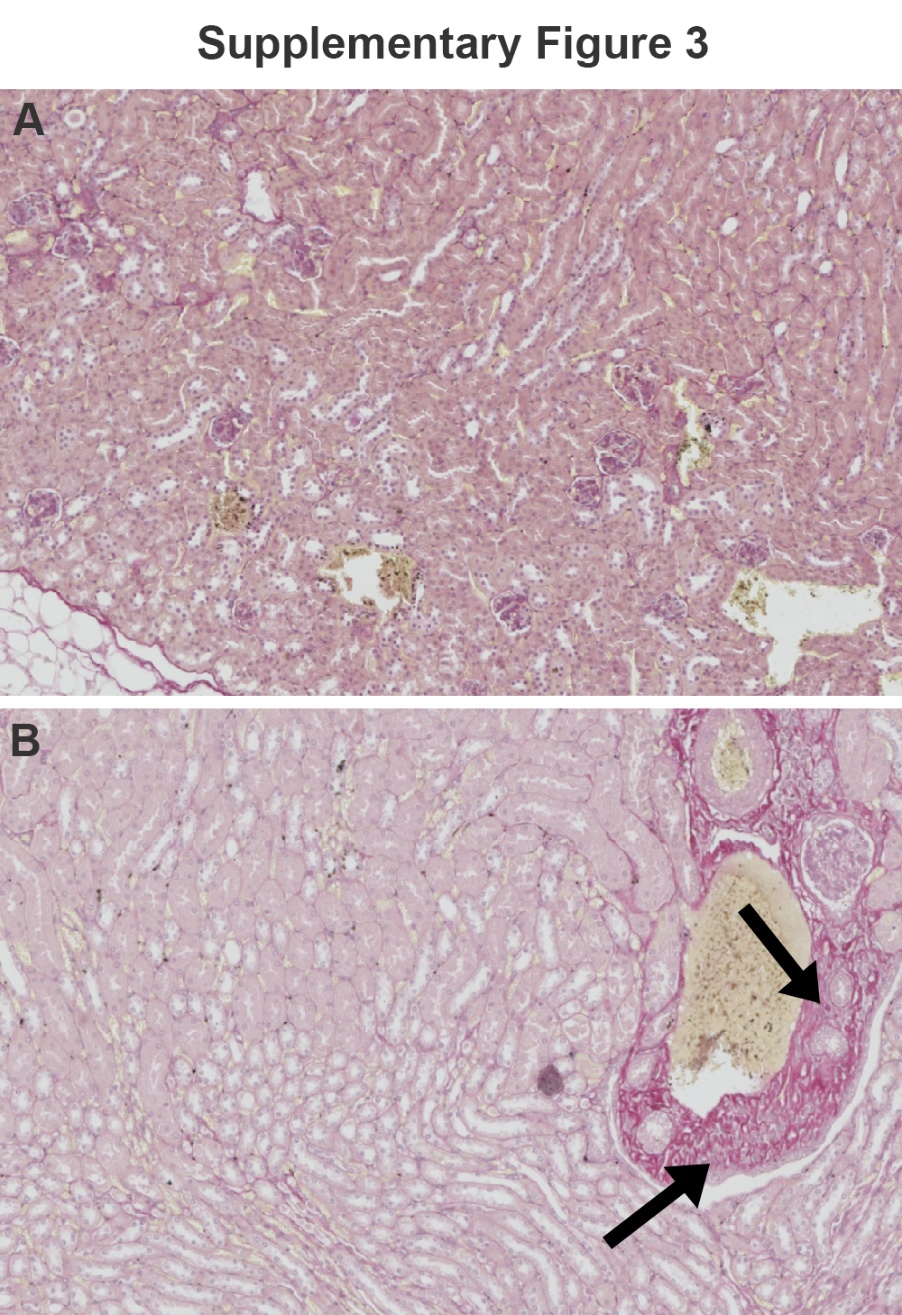


**Supplementary Fig 3. Red Sirius staining of kidney for fibrosis analysis.** WT and C3KO mice were inoculated with PBS (control; CTRL) or 10^8^ *L interrogans* serovar Copenhageni strain FIOCRUZ L1-130 (LIC) (i/p) and monitored for 15, 30, 60, 90, and 180 days post-infection (d.p.i.). Here, we show sections of staining from a healthy C3KO mouse kidney (**A**) and an LIC-infected C3KO mouse kidney (**B**) from the 30 d.p.i. group. The collagen deposition is marked with a red color and indicated by the black arrows. Images are shown at 200x magnification. Mice were obtained from the Animal Care Unit from ICB-USP.
