# Supplementary material for: Complement C3 deficiency enhances renal leptospiral load and inflammation while impairing T cell differentiation during chronic Leptospira interrogans infection
Source: Infect Immun. 2025 Nov 18;93(12):e00398-25. doi: 10.1128/iai.00398-25 (PMC12707143; doi:10.1128/iai.00398-25)
Supplement: Figure S4 — Fibrosis genes analyzed. [file iai.00398-25-s0004.docx]

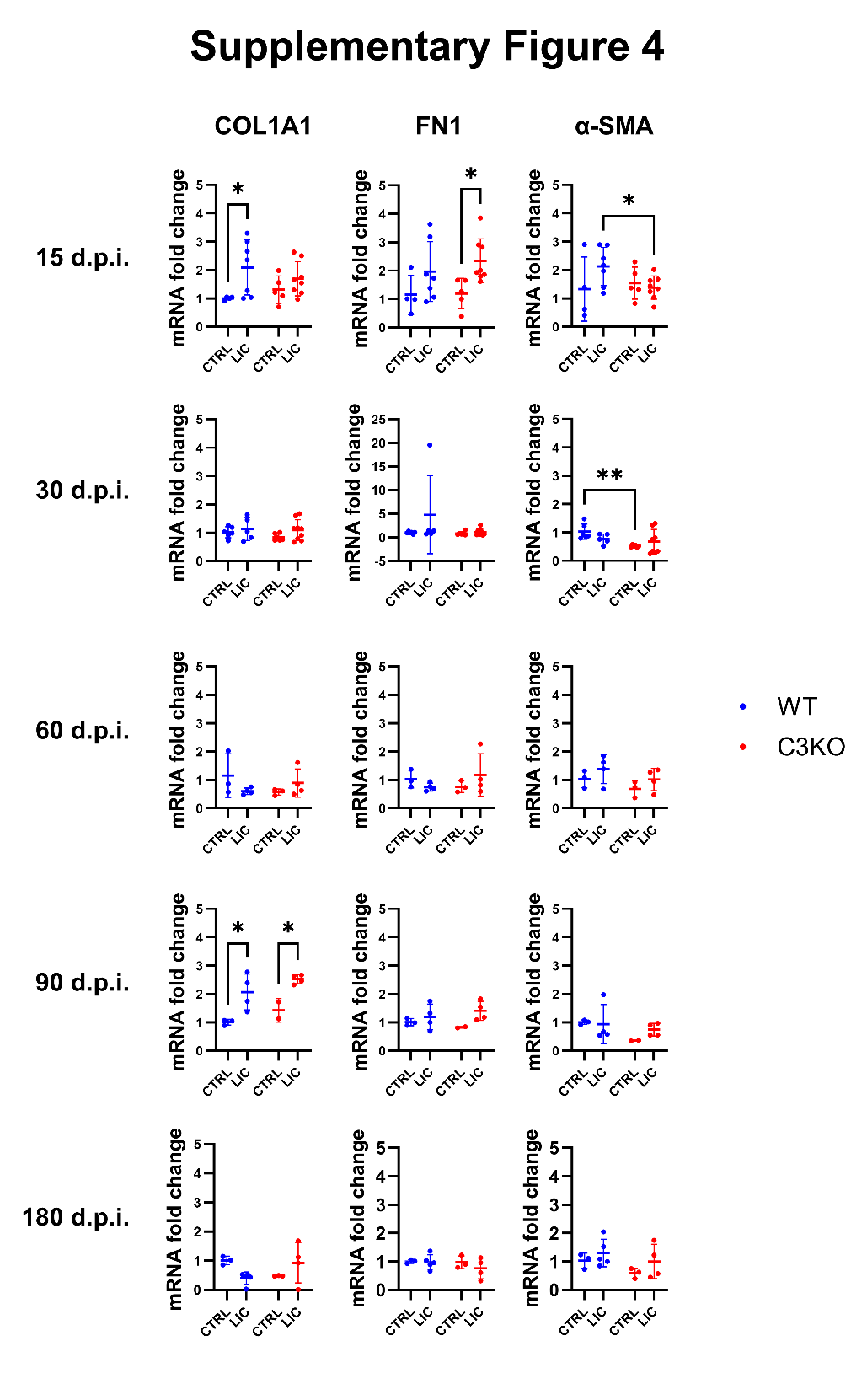
**Supplementary Fig 4. Gene expression of fibrosis-related genes.** WT and C3KO mice were inoculated with PBS (control; CTRL) or 10^8^ *L interrogans* serovar Copenhageni strain FIOCRUZ L1-130 (LIC) (i/p). After 15, 30, 60, 90 and 180 days, total RNA was extracted from kidney samples to build cDNA from mRNA. We quantified the expression of COL1A1, FN1 and α-SMA through ΔΔCt comparative method, keeping β-actin as the endogenous control. A two-way ANOVA was performed, followed by Tukey’s post-hoc test. Significance was set at α = 0.05. **p* < 0,05; ***p* < 0,01. Mice were obtained from the Animal Care Unit from ICB-USP.
