# Supplementary material for: Complement C3 deficiency enhances renal leptospiral load and inflammation while impairing T cell differentiation during chronic Leptospira interrogans infection
Source: Infect Immun. 2025 Nov 18;93(12):e00398-25. doi: 10.1128/iai.00398-25 (PMC12707143; doi:10.1128/iai.00398-25)
Supplement: Figure S5 — ELISA for immunoglobulin subtypes after 30 d.p.i. [file iai.00398-25-s0005.docx]

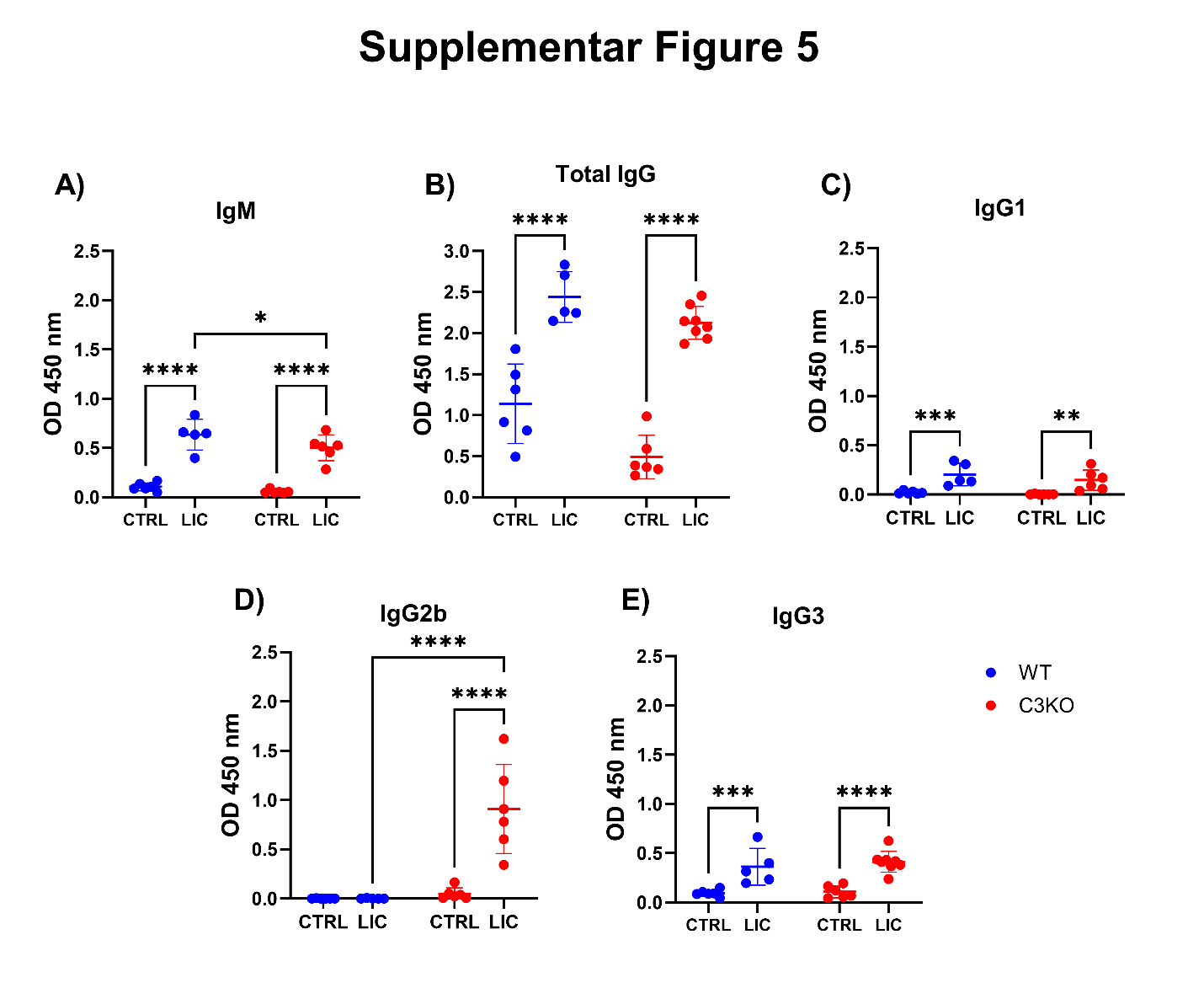


**Supplementary Fig 5. Quantification of specific antibodies against LIC.** WT or C3KO mice were inoculated with PBS (control, CTRL) or 10^8^ *L interrogans* serovar Copenhageni strain FIOCRUZ L1-130 (LIC) (i/p). After 30 d.p.i., serum was obtained and levels of specific IgM, total IgG, and other IgG subclasses anti-LIC were quantified by ELISA. Wells were coated with 10^6^ heat-killed LIC and incubated with diluted mice serum (1:100). Secondary antibodies against **(A)** IgM, **(B)** total IgG, **(C)** IgG1, **(D)** IgG2b, and **(e)** IgG3 were used (1:5000). Each dot represents one animal, (n = 5-6 for PBS groups; n = 5-10 for infected groups). Statistical analysis was performed using two-way ANOVA followed by Tukey’s test, with familiar α of 0.95. *p-*values: *< 0.05; ** *p* < 0.01, ****p*< 0.001; *****p*< 0.0001.
