# Supplementary material for: Complement C3 deficiency enhances renal leptospiral load and inflammation while impairing T cell differentiation during chronic Leptospira interrogans infection
Source: Infect Immun. 2025 Nov 18;93(12):e00398-25. doi: 10.1128/iai.00398-25 (PMC12707143; doi:10.1128/iai.00398-25)
Supplement: Figure S6 — Spleen cytometry analysis. [file iai.00398-25-s0006.docx]

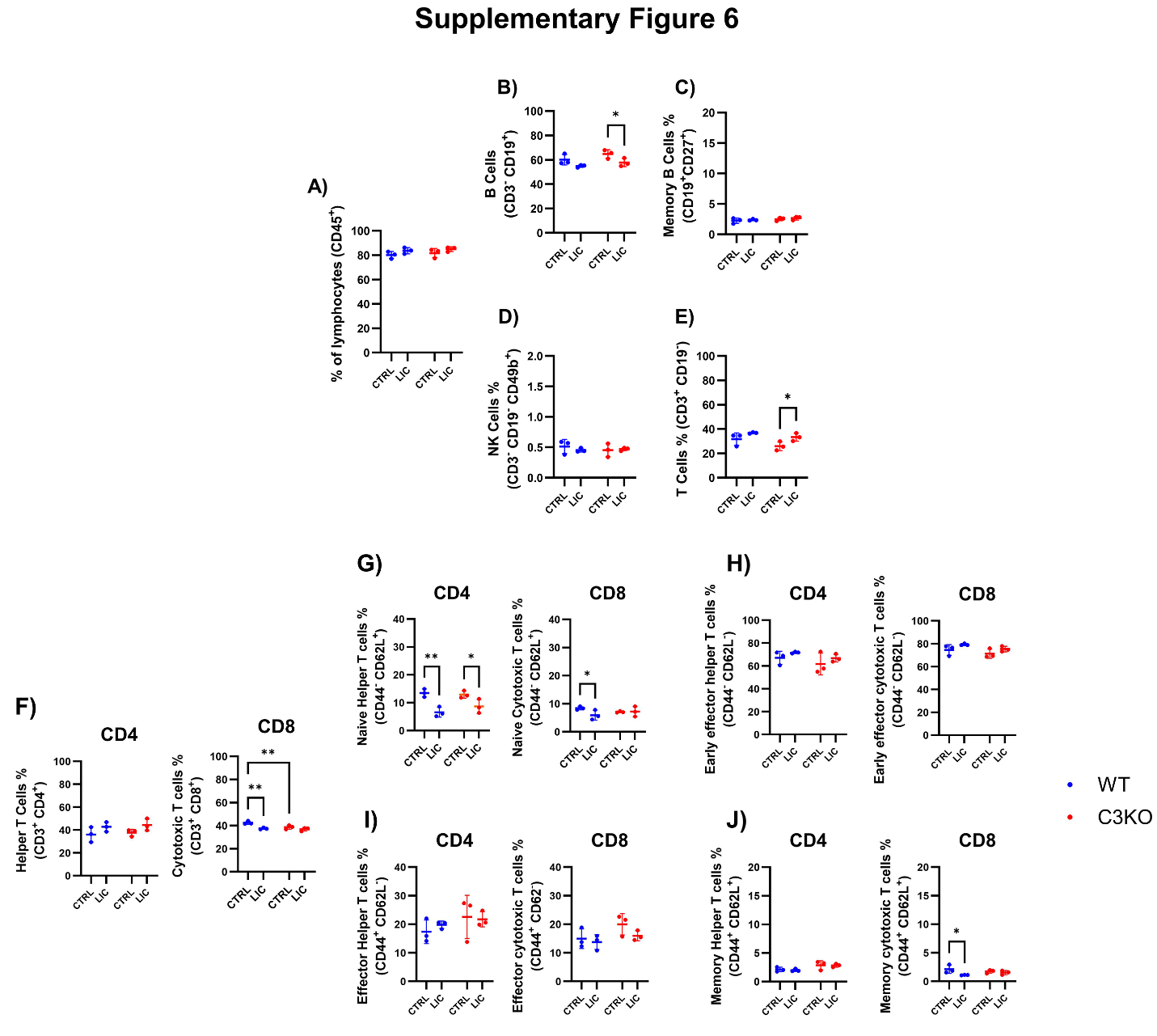


**Supplementary Fig 6. Immune cell populations in the spleen.** WT or C3KO mice were inoculated with PBS (control; CTRL) or with 10^8^ *L interrogans* serovar Copenhageni strain FIOCRUZ L1-130 (LIC) (i/p). Graphs represent the percentage of CD45^+^ T and B lymphocytes, their subpopulations, and NK cells after 30 days post-infection. Each dot represents one animal (n =3 per group). Statistical analysis was performed using two-way ANOVA, followed by Tukey’s test, with familiar α of 0.95. *p-*values: *< 0.05; **< 0.01; ns = non-significant. Mice were obtained from the Animal Care Unit from UTHSC.
