# Supplementary material for: Complement C3 deficiency enhances renal leptospiral load and inflammation while impairing T cell differentiation during chronic Leptospira interrogans infection
Source: Infect Immun. 2025 Nov 18;93(12):e00398-25. doi: 10.1128/iai.00398-25 (PMC12707143; doi:10.1128/iai.00398-25)
Supplement: Figure S7 — Proteome analysis of the serum. [file iai.00398-25-s0007.docx]

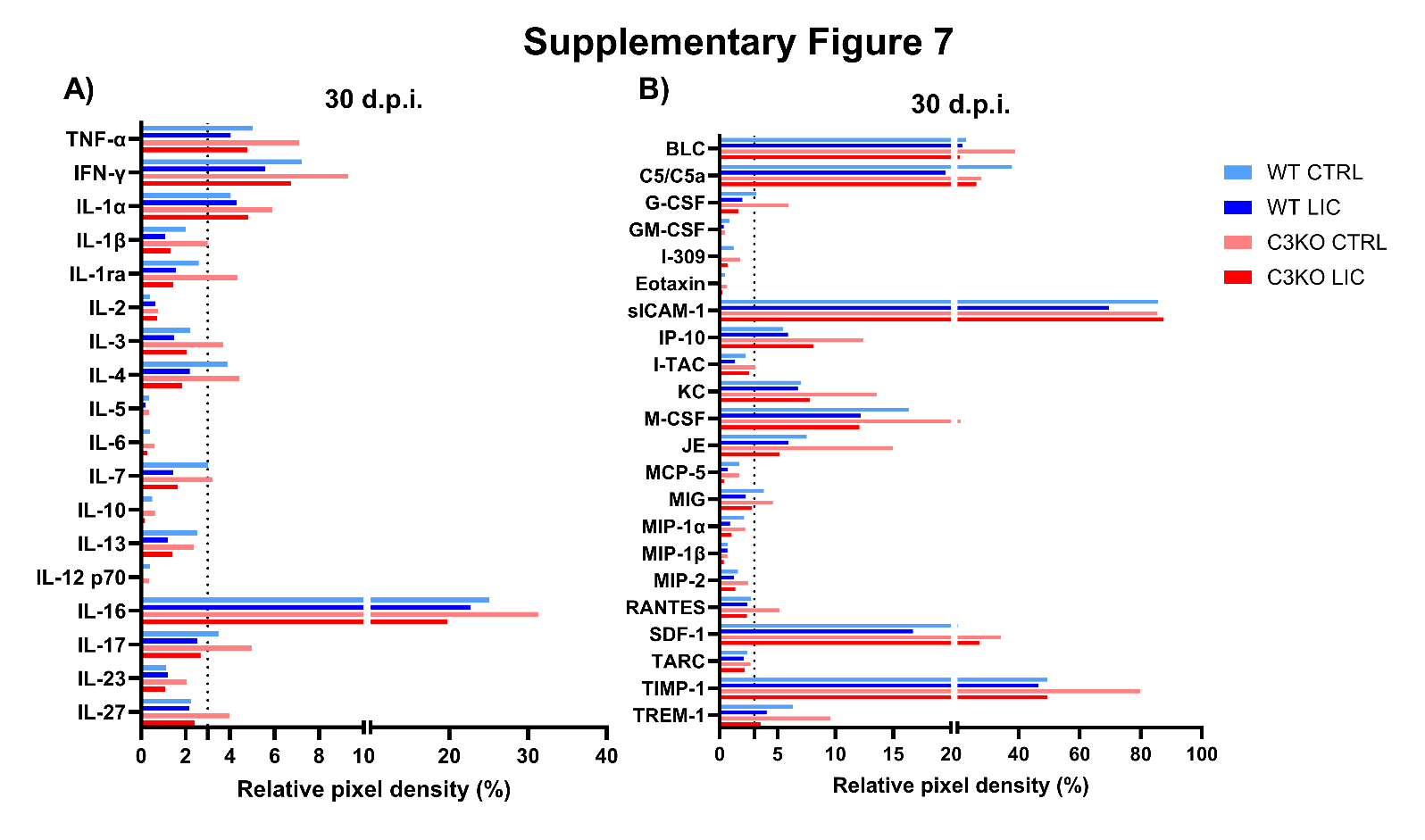


**Supplementary Fig 7. Serum cytokine and acute-phase protein expression in LIC-infected mice at 30 d.p.i.** Chemiluminescence was measured on the pixel density mean of two values using Quickspot software (ARY006 kit). Values were normalized by subtracting control reference values (PBS) and calibrating with six positive control values. Results were considered different if there was a ± 3% difference. Cytokine levels below 3% (dotted line) were considered 0%. Mice were obtained from the Animal Care Unit from UTHSC. CTRL group (n=3) and LIC-infected group (n=5).
