# Supplementary material for: Complement C3 deficiency enhances renal leptospiral load and inflammation while impairing T cell differentiation during chronic Leptospira interrogans infection
Source: Infect Immun. 2025 Nov 18;93(12):e00398-25. doi: 10.1128/iai.00398-25 (PMC12707143; doi:10.1128/iai.00398-25)
Supplement: Table S1 — Antibody panel for cell cytometry. [file iai.00398-25-s0008.docx]

**Supplementary Table 1. Panel of antibodies used for the lymphoid cell cytometry**

| **Cell marker** | **Fluorophore** | **Company** |
| --- | --- | --- |
| **CD45** | **Brilliant violet 605** | **Biolegend** |
| **CD3** | **Violet fluor 450** | **CYTEK** |
| **CD19** | **Brilliant violet 785** | **Biolegend** |
| **CD49b** | **Alexa 700** | **Biolegend** |
| **CD4** | **PE** | **CYTEK** |
| **CD8** | **APC-Cy7** | **CYTEK** |
| **CD44** | **APC** | **Biolegend** |
| **CD62L** | **PE-Cy7** | **CYTEK** |
| **CD27** | **FITC** | **Biolegend** |
